# *catena*-Poly[[[aqua­chlorido­manganese(II)]-bis­[μ-1,1′-(oxydi-*p*-phenyl­ene)di-1*H*-imidazole-κ^2^ *N* ^3^:*N* ^3′^]] chloride dimethyl­formamide mono­solvate monohydrate]

**DOI:** 10.1107/S1600536810054383

**Published:** 2011-01-15

**Authors:** Xiao-Long Mu

**Affiliations:** a College of Chemistry and Materials Science, Anhui Normal University, Wuhu 241000, People’s Republic of China

## Abstract

The title coordination polymer, {[MnCl(C_18_H_14_N_4_O)_2_(H_2_O)]Cl·C_3_H_7_NO·H_2_O}_*n*_, obtained by the solvothermal reaction of BIDPE and manganese(II) salt in H_2_O/DMF (DMF is dimethyl­formamide), is composed of a chain of [Mn_2_(BIDPE)_2_] [BIDPE is 1,1′-(oxydi-*p*-phenyl­ene)di-1*H*-imidazole] metallocyclic rings that exhibit inversion symmetry. The coordination about the Mn(II) ions is distorted octahedral with a MnClN_4_O coordination set.  In the crystal, the polymeric chains are linked by O—H⋯Cl hydrogen bonds, forming a two-dimensional network parallel to (100). A number of C—H⋯Cl and C—H⋯O inter­actions are also present.

## Related literature

For potential applications of metal-organic frameworks, see: Feng *et al.* (2009 ▶); Bauer *et al.* (2007 ▶); Kumagai *et al.* (2002 ▶); Bi *et al.* (2009 ▶); Reddy *et al.* (2010 ▶); Cho *et al.* (2006 ▶); Maji *et al.* (2005 ▶); Zhang *et al.* (2009 ▶). For the synthesis of the 4,4′-bis­(imidazol-1-yl) diphenyl ether (BIDPE) ligand, see: Hu *et al.* (2010 ▶).
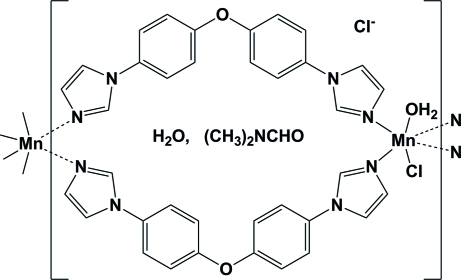


## Experimental

### 

#### Crystal data

[MnCl(C_18_H_14_N_4_O)_2_(H_2_O)]Cl·C_3_H_7_NO·H_2_O
*M* *_r_* = 839.63
Triclinic, 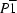

*a* = 12.6167 (14) Å
*b* = 12.6183 (14) Å
*c* = 13.5274 (15) Å
α = 74.801 (2)°
β = 69.388 (2)°
γ = 85.582 (2)°
*V* = 1944.9 (4) Å^3^
*Z* = 2
Mo *K*α radiation
μ = 0.53 mm^−1^
*T* = 273 K
0.32 × 0.30 × 0.29 mm

#### Data collection

Bruker SMART CCD area-detector diffractometer
Absorption correction: multi-scan (*SADABS*; Bruker, 2001 ▶) *T*_min_ = 0.843, *T*_max_ = 0.857
10456 measured reflections
7427 independent reflections
5475 reflections with *I* > 2σ(*I*)
*R*_int_ = 0.047

#### Refinement

*R*[*F* ^2^ > 2σ(*F* ^2^)] = 0.048
*wR*(*F* ^2^) = 0.133
*S* = 1.08
7427 reflections
517 parameters
6 restraints
H atoms treated by a mixture of independent and constrained refinement
Δρ_max_ = 0.55 e Å^−3^
Δρ_min_ = −0.42 e Å^−3^

### 

Data collection: *SMART* (Bruker, 2007 ▶); cell refinement: *SAINT* (Bruker, 2007 ▶); data reduction: *SAINT*; program(s) used to solve structure: *SHELXS97* (Sheldrick, 2008 ▶); program(s) used to refine structure: *SHELXL97* (Sheldrick, 2008 ▶); molecular graphics: *SHELXTL* (Sheldrick, 2008 ▶); software used to prepare material for publication: *SHELXTL*.

## Supplementary Material

Crystal structure: contains datablocks global, I. DOI: 10.1107/S1600536810054383/su2233sup1.cif

Structure factors: contains datablocks I. DOI: 10.1107/S1600536810054383/su2233Isup2.hkl

Additional supplementary materials:  crystallographic information; 3D view; checkCIF report

## Figures and Tables

**Table 1:** Hydrogen-bond geometry (Å, °)

*D*—H⋯*A*	*D*—H	H⋯*A*	*D*⋯*A*	*D*—H⋯*A*
O3—H3*A*⋯Cl2^i^	0.86 (2)	2.29 (2)	3.1306 (19)	166 (2)
O3—H3*B*⋯Cl2	0.85 (2)	2.27 (2)	3.093 (2)	162 (3)
O5—H5*A*⋯Cl1^ii^	0.89 (2)	2.81 (5)	3.282 (3)	114 (4)
O5—H5*B*⋯Cl1^iii^	0.95 (3)	2.48 (4)	3.316 (4)	148 (5)
C17—H17⋯Cl2^i^	0.93	2.67	3.553 (3)	159
C18—H18⋯O5^ii^	0.93	2.50	3.418 (5)	170
C23—H23⋯O4^iv^	0.93	2.47	3.270 (6)	145
C35—H35⋯Cl1^v^	0.93	2.82	3.398 (3)	121

Symmetry codes: (i) 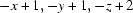; (ii) 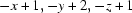; (iii) 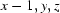; (iv) 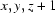; (v) 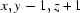.

**Fig. 1.**
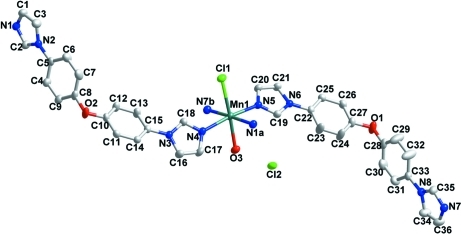
The molecular structure of the title compound, showing the asymmetric unit and key symmetry-related atoms. Displacement ellipsoids are drawn at the 30% probability level. Symmetry codes: (a) x, -1 - y, 1 + z; (b) x, 1 + y, -1 + z.

**Fig. 2.**
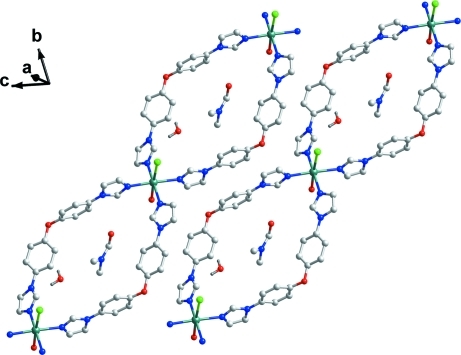
A view of the necklace-like one-dimensional chains of the title compound, formed by BIDPE and MnII ions. The water and DMF molecules are located in the [Mn2(BIDPE)2] metallocyclic ring cavities.

**Fig. 3.**
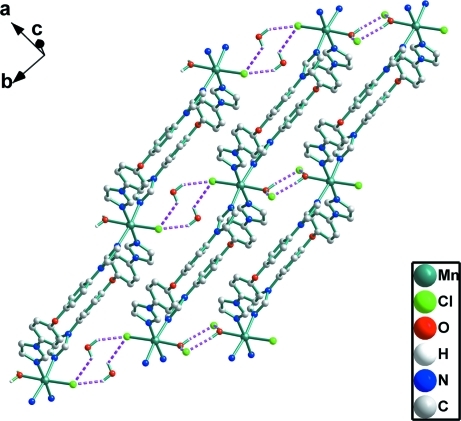
A view of the two-dimensional hydrogen bonding network in the title compound (see Table 1 for details).

## supplementary crystallographic information

### Comment

The design and construction of metal-organic frameworks (MOF's) from various molecular building blocks is of great interest due to their novel architectures and potential applications in, for example, photochemical areas (Feng *et al.*, 2009; Bauer *et al.*, 2007), molecular magnetism (Kumagai *et al.*, 2002; Bi *et al.*, 2009), heterogeneous catalysis (Reddy *et al.*, 2010; Cho *et al.*, 2006), and molecular sorption (Maji *et al.*, 2005; Zhang *et al.*, 2009). We recently designed and synthesized 4,4'-bis(imidazol-1-yl) diphenyl ether (BIDPE), a V-shaped imidazole molecule which can be regarded as a semi-flexible ligand (Hu *et al.*, 2010). To test the ability of this ligand to give new architectures and topologies its reaction with a bivalent manganese(II) salt was studied solvothermally, and resulted in the synthesize of the new title coordination polymer.

The asymmetric unit of the title compound consists of one Mn^II ^ion, two BIDPE molecules, one coordinated Cl^-^ anion and water molecule, and one lattice Cl^-^ anion, one lattice water, and one DMF molecule (Fig. 1). The Mn^II ^ion is six-coordinate with a distorted octahedral geometry. It is coordinted to four N atoms from four BIDPE ligands, one Cl^-^ anion, and one O atom from a water molecule. The Mn—N bond lengths vary from 2.227 (2) to 2.272 (2) Å, which is within the range reported for octahedral manganese(II) complexes.

Neighbouring Mn^II^ ions are linked by BIDPE ligands and Cl^-^ anions to form an infinitely necklace-like one-dimensional chain. Two BIDPE ligands connect two Mn^II^ atoms to achieve a 32-membered [Mn_2_(BIDPE)_2_] macrocycle, exhibiting maximum dimensions of  15.90 × 10.88 Å (corresponding to the Mn···Mn distance and O···O separation, respectively). The angles N1–Mn1–N5 and N4–Mn1–N7 are 89.60 (7)° and 88.75 (8)°, respectively. The lattice water and DMF molecules are found located in the large [Mn_2_(BIDPE)_2_] metallocyclic ring cavities (Fig. 2).

Further inspection shows that the coordinated and lattice water molecules and the Cl^-^ anions, are linked by strong O-H···Cl hydrogen bonds (Table 1 and Fig. 3). These interactions are also available for increasing the stability of the whole crystal structure. This extension of the structure into a two-dimensional network is accomplished by O-H···Cl hydrogen bonding, involving the coordinated Cl atoms and the water molecule of crystallization. There are also a number of C-H···O and C-H···Cl interactions present in the crystal structure (Table 1).

### Experimental

A mixture of MnCl_2_.4H_2_O (19.8 mg, 0.1 mmol), BIDPE (56.8 mg, 0.1 mmol) was dissolved in10 ml of DMF/H_2_O(1:4, *v*/*v*). The final mixture was placed in a Parr Teflon-lined stainless steel vessel (10 ml) under autogenous pressure and was heated at 363 K for 3 d. The clear solution obtained was volatilized over a period of a few weeks. A large quantity of colourless block-like crystals were obtained, which were washed with the mother liquor, and dried under ambient conditions (Yield: 53% based on Mn).

### Refinement

The water H-atoms were located from a Fourier differnce map and were refined with distance restraintes of O-H = 0.85 (2) Å, and H···H = 1.45 (2) Å with *U*_iso_(H) = 1.5*U*_eq_(O). The C-bound H-atoms were placed in geometrically idealized positions and treated as riding: C—H = 0.93 and 0.96 Å for CH and CH_3_ H-atoms, respectively, with *U*_iso_(H) = k × *U*_eq_(C), where k = 1.5 for CH_3_ H-atoms, and k = 1.2 for all other H-atoms.

### Crystal data

**Table:**

[MnCl(C_18_H_14_N_4_O)_2_(H_2_O)]Cl·C_3_H_7_NO·H_2_O	*Z* = 2
*M**_r_* = 839.63	*F*(000) = 870
Triclinic, *P*1	*D*_x_ = 1.434 Mg m^−^^3^
Hall symbol: -P 1	Mo *K*α radiation, λ = 0.71073 Å
*a* = 12.6167 (14) Å	Cell parameters from 4541 reflections
*b* = 12.6183 (14) Å	θ = 2.3–27.4°
*c* = 13.5274 (15) Å	µ = 0.53 mm^−^^1^
α = 74.801 (2)°	*T* = 273 K
β = 69.388 (2)°	Block, colourless
γ = 85.582 (2)°	0.32 × 0.30 × 0.29 mm
*V* = 1944.9 (4) Å^3^	

### Data collection

**Table:**

Bruker SMART CCD area-detector diffractometer	7427 independent reflections
Radiation source: fine-focus sealed tube	5475 reflections with *I* > 2σ(*I*)
graphite	*R*_int_ = 0.047
phi and ω scans	θ_max_ = 26.0°, θ_min_ = 1.7°
Absorption correction: multi-scan (*SADABS*; Bruker, 2001)	*h* = −15→14
*T*_min_ = 0.843, *T*_max_ = 0.857	*k* = −12→15
10456 measured reflections	*l* = −16→16

### Refinement

**Table:**

Refinement on *F*^2^	Primary atom site location: structure-invariant direct methods
Least-squares matrix: full	Secondary atom site location: difference Fourier map
*R*[*F*^2^ > 2σ(*F*^2^)] = 0.048	Hydrogen site location: inferred from neighbouring sites
*wR*(*F*^2^) = 0.133	H atoms treated by a mixture of independent and constrained refinement
*S* = 1.08	*w* = 1/[σ^2^(*F*_o_^2^) + (0.0723*P*)^2^] where *P* = (*F*_o_^2^ + 2*F*_c_^2^)/3
7427 reflections	(Δ/σ)_max_ < 0.001
517 parameters	Δρ_max_ = 0.55 e Å^−^^3^
6 restraints	Δρ_min_ = −0.42 e Å^−^^3^

### Special details

**Table:**

Geometry. Bond distances, angles etc. have been calculated using the rounded fractional coordinates. All su's are estimated from the variances of the (full) variance-covariance matrix. The cell esds are taken into account in the estimation of distances, angles and torsion angles
Refinement. Refinement of *F*^2^ against ALL reflections. The weighted *R*-factor *wR* and goodness of fit *S* are based on *F*^2^, conventional *R*-factors *R* are based on *F*, with *F* set to zero for negative *F*^2^. The threshold expression of *F*^2^ > σ(*F*^2^) is used only for calculating *R*-factors(gt) *etc*. and is not relevant to the choice of reflections for refinement. *R*-factors based on *F*^2^ are statistically about twice as large as those based on *F*, and *R*- factors based on ALL data will be even larger.

### Fractional atomic coordinates and isotropic or equivalent isotropic displacement parameters (Å^2^)

**Table:**

	*x*	*y*	*z*	*U*_iso_*/*U*_eq_	
Mn1	0.77799 (3)	0.67916 (3)	0.73266 (3)	0.0441 (1)	
Cl1	0.96491 (6)	0.77037 (6)	0.59685 (5)	0.0610 (2)	
O1	0.8288 (2)	−0.10963 (14)	0.97544 (16)	0.0760 (8)	
O2	0.67208 (19)	1.45186 (14)	0.49834 (14)	0.0688 (7)	
O3	0.60727 (16)	0.60189 (14)	0.84342 (14)	0.0557 (6)	
N1	0.83399 (18)	1.64559 (16)	−0.12340 (16)	0.0505 (7)	
N2	0.83490 (17)	1.59740 (15)	0.04514 (15)	0.0465 (7)	
N3	0.65781 (17)	1.01475 (15)	0.72708 (16)	0.0456 (6)	
N4	0.69914 (18)	0.83954 (15)	0.76500 (16)	0.0519 (7)	
N5	0.83206 (16)	0.50934 (15)	0.71176 (15)	0.0465 (7)	
N6	0.85574 (16)	0.33001 (15)	0.74462 (15)	0.0416 (6)	
N7	0.71164 (18)	−0.29343 (16)	1.59221 (16)	0.0496 (7)	
N8	0.69930 (17)	−0.24606 (16)	1.42776 (16)	0.0467 (7)	
C1	0.9383 (2)	1.6657 (2)	−0.1235 (2)	0.0624 (10)	
C2	0.7740 (2)	1.6045 (2)	−0.0203 (2)	0.0544 (9)	
C3	0.9399 (2)	1.6361 (3)	−0.0219 (2)	0.0669 (11)	
C4	0.6801 (2)	1.5476 (2)	0.2179 (2)	0.0559 (9)	
C5	0.7940 (2)	1.55776 (18)	0.16115 (19)	0.0465 (8)	
C6	0.8683 (3)	1.5293 (3)	0.2169 (2)	0.0676 (11)	
C7	0.8280 (3)	1.4919 (3)	0.3293 (2)	0.0731 (11)	
C8	0.7149 (3)	1.48293 (19)	0.3842 (2)	0.0549 (9)	
C9	0.6396 (3)	1.5097 (2)	0.3302 (2)	0.0589 (9)	
C10	0.6700 (2)	1.34167 (18)	0.54990 (19)	0.0469 (8)	
C11	0.6346 (2)	1.31692 (19)	0.66134 (19)	0.0445 (8)	
C12	0.6999 (2)	1.2599 (2)	0.4957 (2)	0.0577 (9)	
C13	0.6945 (2)	1.1517 (2)	0.5548 (2)	0.0571 (9)	
C14	0.63090 (19)	1.20996 (18)	0.72022 (19)	0.0425 (7)	
C15	0.66031 (19)	1.12626 (18)	0.66690 (19)	0.0423 (7)	
C16	0.5957 (2)	0.9700 (2)	0.8342 (2)	0.0620 (9)	
C17	0.6216 (3)	0.8632 (2)	0.8561 (2)	0.0636 (10)	
C18	0.7187 (2)	0.93270 (19)	0.6901 (2)	0.0509 (8)	
C19	0.8186 (2)	0.41719 (19)	0.78742 (19)	0.0456 (8)	
C20	0.8798 (2)	0.4799 (2)	0.6154 (2)	0.0519 (8)	
C21	0.8948 (2)	0.3713 (2)	0.6331 (2)	0.0536 (9)	
C22	0.84856 (19)	0.21715 (18)	0.80412 (19)	0.0432 (8)	
C23	0.7961 (3)	0.1899 (2)	0.9157 (2)	0.0779 (12)	
C24	0.7895 (3)	0.0817 (2)	0.9744 (2)	0.0825 (12)	
C25	0.8900 (2)	0.13555 (19)	0.7517 (2)	0.0462 (8)	
C26	0.8822 (2)	0.0267 (2)	0.8120 (2)	0.0497 (8)	
C27	0.8334 (2)	0.0012 (2)	0.9223 (2)	0.0541 (9)	
C28	0.7946 (3)	−0.1378 (2)	1.0886 (2)	0.0593 (10)	
C29	0.8731 (3)	−0.1478 (4)	1.1373 (3)	0.1008 (16)	
C30	0.6857 (3)	−0.1647 (3)	1.1507 (3)	0.0754 (12)	
C31	0.6537 (3)	−0.1984 (3)	1.2625 (3)	0.0693 (11)	
C32	0.8403 (3)	−0.1825 (4)	1.2488 (3)	0.1027 (16)	
C33	0.7314 (2)	−0.20775 (19)	1.3121 (2)	0.0476 (8)	
C34	0.5980 (2)	−0.2919 (3)	1.5001 (3)	0.0744 (13)	
C35	0.7643 (2)	−0.2506 (2)	1.4885 (2)	0.0555 (9)	
C36	0.6072 (2)	−0.3191 (3)	1.5991 (2)	0.0682 (10)	
O4	0.7709 (4)	0.3324 (4)	0.0926 (4)	0.168 (2)	
N9	0.6951 (4)	0.1770 (4)	0.2291 (4)	0.1182 (19)	
C37	0.8015 (5)	0.1377 (5)	0.2378 (5)	0.170 (4)	
C38	0.5998 (6)	0.1098 (5)	0.3003 (5)	0.170 (3)	
C39	0.6847 (6)	0.2690 (5)	0.1628 (5)	0.142 (3)	
O5	0.0859 (3)	1.0060 (3)	0.5680 (3)	0.1261 (14)	
Cl2	0.53260 (6)	0.35717 (5)	0.92795 (5)	0.0602 (2)	
H1	0.99960	1.69570	−0.18530	0.0750*	
H2	0.69830	1.58280	0.00450	0.0650*	
H3	1.00170	1.64100	−0.00090	0.0800*	
H3A	0.563 (2)	0.622 (2)	0.8997 (19)	0.0840*	
H3B	0.598 (3)	0.5332 (15)	0.853 (2)	0.0840*	
H4	0.62940	1.56650	0.18050	0.0670*	
H6	0.94590	1.53510	0.17900	0.0810*	
H7	0.87840	1.47300	0.36710	0.0880*	
H9	0.56210	1.50250	0.36840	0.0710*	
H11	0.61310	1.37280	0.69730	0.0530*	
H12	0.72360	1.27710	0.42000	0.0690*	
H13	0.71400	1.09580	0.51870	0.0690*	
H14	0.60870	1.19350	0.79590	0.0510*	
H16	0.54520	1.00650	0.88260	0.0740*	
H17	0.59120	0.81280	0.92320	0.0760*	
H18	0.76920	0.94090	0.61910	0.0610*	
H19	0.78720	0.41230	0.86190	0.0550*	
H20	0.89910	0.52870	0.54670	0.0620*	
H21	0.92550	0.33170	0.58070	0.0640*	
H23	0.76510	0.24460	0.95170	0.0940*	
H24	0.75490	0.06380	1.04990	0.0990*	
H25	0.92330	0.15280	0.67610	0.0550*	
H26	0.91060	−0.02870	0.77650	0.0600*	
H29	0.94890	−0.13140	1.09540	0.1210*	
H30	0.63170	−0.16050	1.11760	0.0910*	
H31	0.57800	−0.21480	1.30440	0.0830*	
H32	0.89470	−0.18870	1.28170	0.1240*	
H34	0.53450	−0.30220	1.48370	0.0890*	
H35	0.83920	−0.22590	1.45970	0.0670*	
H36	0.54970	−0.35110	1.66360	0.0820*	
H37A	0.85450	0.13770	0.16660	0.2550*	
H37B	0.79180	0.06440	0.28380	0.2550*	
H37C	0.82970	0.18470	0.26880	0.2550*	
H38A	0.53170	0.14790	0.29720	0.2550*	
H38B	0.60040	0.09310	0.37350	0.2550*	
H38C	0.60270	0.04280	0.27840	0.2550*	
H39	0.61220	0.29140	0.16490	0.1710*	
H5A	0.020 (3)	1.036 (4)	0.569 (5)	0.1890*	
H5B	0.075 (4)	0.9290 (15)	0.588 (5)	0.1890*	

### Atomic displacement parameters (Å^2^)

**Table:**

	*U*^11^	*U*^22^	*U*^33^	*U*^12^	*U*^13^	*U*^23^
Mn1	0.0548 (2)	0.0355 (2)	0.0343 (2)	0.0009 (2)	−0.0104 (2)	−0.0032 (2)
Cl1	0.0563 (4)	0.0623 (4)	0.0507 (4)	−0.0080 (3)	−0.0080 (3)	−0.0035 (3)
O1	0.1391 (19)	0.0409 (10)	0.0501 (12)	0.0153 (11)	−0.0398 (12)	−0.0088 (8)
O2	0.1201 (17)	0.0417 (10)	0.0388 (10)	0.0261 (10)	−0.0261 (10)	−0.0092 (8)
O3	0.0631 (12)	0.0446 (10)	0.0442 (10)	−0.0052 (9)	0.0009 (8)	−0.0111 (8)
N1	0.0627 (14)	0.0466 (11)	0.0370 (11)	0.0064 (10)	−0.0129 (10)	−0.0093 (9)
N2	0.0541 (12)	0.0433 (11)	0.0392 (11)	0.0091 (9)	−0.0153 (10)	−0.0088 (9)
N3	0.0521 (12)	0.0367 (10)	0.0414 (11)	0.0039 (9)	−0.0115 (9)	−0.0061 (8)
N4	0.0667 (14)	0.0382 (11)	0.0427 (12)	0.0023 (10)	−0.0131 (10)	−0.0051 (9)
N5	0.0520 (12)	0.0410 (11)	0.0377 (11)	0.0012 (9)	−0.0075 (9)	−0.0065 (9)
N6	0.0436 (11)	0.0415 (10)	0.0360 (10)	0.0045 (8)	−0.0110 (8)	−0.0083 (8)
N7	0.0559 (13)	0.0480 (11)	0.0424 (12)	0.0009 (10)	−0.0155 (10)	−0.0093 (9)
N8	0.0494 (12)	0.0455 (11)	0.0468 (12)	0.0013 (9)	−0.0207 (10)	−0.0086 (9)
C1	0.0549 (17)	0.0725 (18)	0.0451 (16)	0.0057 (14)	−0.0078 (12)	−0.0047 (13)
C2	0.0600 (16)	0.0605 (16)	0.0391 (14)	−0.0027 (13)	−0.0164 (12)	−0.0065 (12)
C3	0.0534 (17)	0.085 (2)	0.0570 (18)	0.0036 (15)	−0.0198 (14)	−0.0089 (15)
C4	0.0616 (17)	0.0607 (16)	0.0427 (14)	0.0002 (13)	−0.0217 (13)	−0.0030 (12)
C5	0.0632 (16)	0.0364 (12)	0.0405 (13)	0.0110 (11)	−0.0204 (12)	−0.0101 (10)
C6	0.0632 (18)	0.087 (2)	0.0526 (17)	0.0195 (15)	−0.0246 (14)	−0.0165 (15)
C7	0.083 (2)	0.089 (2)	0.0544 (18)	0.0326 (18)	−0.0382 (17)	−0.0187 (16)
C8	0.088 (2)	0.0369 (13)	0.0369 (13)	0.0153 (13)	−0.0230 (14)	−0.0065 (10)
C9	0.0710 (18)	0.0526 (16)	0.0446 (15)	−0.0004 (13)	−0.0158 (13)	−0.0033 (12)
C10	0.0602 (15)	0.0402 (13)	0.0404 (13)	0.0145 (11)	−0.0220 (12)	−0.0080 (10)
C11	0.0502 (14)	0.0429 (13)	0.0418 (13)	0.0116 (10)	−0.0166 (11)	−0.0152 (10)
C12	0.089 (2)	0.0469 (14)	0.0353 (13)	0.0180 (13)	−0.0226 (13)	−0.0103 (11)
C13	0.086 (2)	0.0427 (13)	0.0432 (14)	0.0161 (13)	−0.0233 (14)	−0.0146 (11)
C14	0.0446 (13)	0.0451 (13)	0.0344 (12)	0.0049 (10)	−0.0116 (10)	−0.0081 (10)
C15	0.0454 (13)	0.0397 (12)	0.0399 (13)	0.0055 (10)	−0.0155 (10)	−0.0072 (10)
C16	0.0716 (18)	0.0495 (15)	0.0473 (15)	0.0047 (13)	−0.0013 (13)	−0.0103 (12)
C17	0.083 (2)	0.0442 (15)	0.0436 (15)	−0.0056 (13)	−0.0059 (14)	0.0021 (12)
C18	0.0585 (15)	0.0408 (13)	0.0435 (14)	0.0051 (11)	−0.0099 (11)	−0.0062 (11)
C19	0.0492 (14)	0.0438 (13)	0.0376 (13)	0.0026 (11)	−0.0074 (10)	−0.0107 (10)
C20	0.0611 (16)	0.0463 (14)	0.0350 (13)	0.0012 (12)	−0.0054 (11)	−0.0036 (11)
C21	0.0671 (17)	0.0480 (14)	0.0368 (13)	0.0043 (12)	−0.0074 (12)	−0.0116 (11)
C22	0.0477 (14)	0.0391 (12)	0.0423 (13)	0.0064 (10)	−0.0176 (11)	−0.0080 (10)
C23	0.134 (3)	0.0428 (15)	0.0407 (15)	0.0193 (17)	−0.0119 (17)	−0.0138 (12)
C24	0.148 (3)	0.0470 (16)	0.0349 (15)	0.0156 (18)	−0.0157 (17)	−0.0068 (12)
C25	0.0493 (14)	0.0463 (13)	0.0414 (13)	0.0062 (11)	−0.0132 (11)	−0.0133 (11)
C26	0.0583 (15)	0.0448 (13)	0.0491 (15)	0.0110 (11)	−0.0198 (12)	−0.0183 (11)
C27	0.0780 (18)	0.0400 (13)	0.0465 (15)	0.0105 (12)	−0.0286 (13)	−0.0076 (11)
C28	0.096 (2)	0.0367 (13)	0.0478 (16)	0.0071 (14)	−0.0328 (16)	−0.0058 (11)
C29	0.082 (2)	0.160 (4)	0.0516 (19)	−0.047 (2)	−0.0217 (18)	0.000 (2)
C30	0.082 (2)	0.089 (2)	0.066 (2)	0.0223 (19)	−0.0447 (19)	−0.0168 (17)
C31	0.0574 (17)	0.090 (2)	0.0640 (19)	0.0093 (15)	−0.0315 (15)	−0.0129 (16)
C32	0.065 (2)	0.185 (4)	0.0519 (19)	−0.045 (2)	−0.0275 (16)	0.004 (2)
C33	0.0572 (15)	0.0385 (12)	0.0489 (15)	0.0020 (11)	−0.0242 (12)	−0.0061 (11)
C34	0.0487 (16)	0.112 (3)	0.0601 (19)	−0.0149 (16)	−0.0154 (14)	−0.0175 (18)
C35	0.0522 (15)	0.0619 (16)	0.0492 (16)	−0.0068 (12)	−0.0206 (13)	−0.0024 (12)
C36	0.0571 (17)	0.093 (2)	0.0488 (17)	−0.0147 (15)	−0.0073 (13)	−0.0191 (15)
O4	0.222 (5)	0.140 (3)	0.162 (4)	−0.001 (3)	−0.062 (3)	−0.076 (3)
N9	0.127 (3)	0.115 (3)	0.144 (4)	0.022 (3)	−0.058 (3)	−0.075 (3)
C37	0.167 (6)	0.189 (6)	0.227 (7)	0.055 (5)	−0.119 (5)	−0.118 (6)
C38	0.183 (6)	0.162 (6)	0.181 (6)	−0.023 (5)	−0.039 (5)	−0.095 (5)
C39	0.174 (6)	0.112 (4)	0.152 (6)	0.013 (4)	−0.042 (5)	−0.076 (4)
O5	0.132 (3)	0.106 (2)	0.111 (2)	−0.0022 (18)	−0.011 (2)	−0.020 (2)
Cl2	0.0623 (4)	0.0513 (4)	0.0494 (4)	−0.0023 (3)	0.0038 (3)	−0.0143 (3)

### Geometric parameters (Å, °)

**Table:**

Mn1—Cl1	2.5535 (9)	C16—C17	1.342 (4)
Mn1—O3	2.263 (2)	C20—C21	1.338 (4)
Mn1—N4	2.264 (2)	C22—C25	1.369 (3)
Mn1—N5	2.257 (2)	C22—C23	1.376 (3)
Mn1—N1^i^	2.227 (2)	C23—C24	1.380 (4)
Mn1—N7^ii^	2.272 (2)	C24—C27	1.360 (4)
O1—C27	1.390 (3)	C25—C26	1.392 (4)
O1—C28	1.389 (3)	C26—C27	1.357 (3)
O2—C8	1.399 (3)	C28—C30	1.349 (5)
O2—C10	1.380 (3)	C28—C29	1.352 (5)
O3—H3B	0.85 (2)	C29—C32	1.371 (5)
O3—H3A	0.86 (2)	C30—C31	1.375 (5)
O4—C39	1.317 (9)	C31—C33	1.352 (5)
O5—H5A	0.88 (5)	C32—C33	1.349 (5)
O5—H5B	0.95 (3)	C34—C36	1.337 (4)
N1—C2	1.313 (3)	C1—H1	0.9300
N1—C1	1.359 (4)	C2—H2	0.9300
N2—C3	1.356 (3)	C3—H3	0.9300
N2—C2	1.345 (3)	C4—H4	0.9300
N2—C5	1.427 (3)	C6—H6	0.9300
N3—C16	1.368 (3)	C7—H7	0.9300
N3—C18	1.338 (3)	C9—H9	0.9300
N3—C15	1.425 (3)	C11—H11	0.9300
N4—C18	1.309 (3)	C12—H12	0.9300
N4—C17	1.365 (3)	C13—H13	0.9300
N5—C20	1.366 (3)	C14—H14	0.9300
N5—C19	1.309 (3)	C16—H16	0.9300
N6—C21	1.376 (3)	C17—H17	0.9300
N6—C22	1.432 (3)	C18—H18	0.9300
N6—C19	1.352 (3)	C19—H19	0.9300
N7—C36	1.347 (4)	C20—H20	0.9300
N7—C35	1.305 (3)	C21—H21	0.9300
N8—C34	1.365 (4)	C23—H23	0.9300
N8—C35	1.339 (3)	C24—H24	0.9300
N8—C33	1.426 (3)	C25—H25	0.9300
N9—C37	1.428 (9)	C26—H26	0.9300
N9—C38	1.416 (9)	C29—H29	0.9300
N9—C39	1.297 (8)	C30—H30	0.9300
C1—C3	1.333 (4)	C31—H31	0.9300
C4—C9	1.381 (3)	C32—H32	0.9300
C4—C5	1.368 (4)	C34—H34	0.9300
C5—C6	1.371 (4)	C35—H35	0.9300
C6—C7	1.382 (3)	C36—H36	0.9300
C7—C8	1.355 (5)	C37—H37C	0.9600
C8—C9	1.364 (5)	C37—H37A	0.9600
C10—C12	1.374 (3)	C37—H37B	0.9600
C10—C11	1.368 (3)	C38—H38A	0.9600
C11—C14	1.370 (3)	C38—H38B	0.9600
C12—C13	1.383 (4)	C38—H38C	0.9600
C13—C15	1.376 (3)	C39—H39	0.9300
C14—C15	1.387 (3)		
			
Cl1—Mn1—O3	175.97 (5)	O1—C27—C26	116.2 (2)
Cl1—Mn1—N4	93.74 (6)	O1—C28—C30	121.0 (3)
Cl1—Mn1—N5	93.94 (6)	O1—C28—C29	119.5 (3)
Cl1—Mn1—N1^i^	94.66 (6)	C29—C28—C30	119.2 (3)
Cl1—Mn1—N7^ii^	88.74 (6)	C28—C29—C32	119.6 (4)
O3—Mn1—N4	85.63 (7)	C28—C30—C31	120.7 (4)
O3—Mn1—N5	86.57 (7)	C30—C31—C33	120.6 (4)
O3—Mn1—N1^i^	89.34 (8)	C29—C32—C33	121.9 (4)
O3—Mn1—N7^ii^	87.27 (7)	N8—C33—C31	120.8 (3)
N4—Mn1—N5	172.07 (8)	N8—C33—C32	121.1 (3)
N1^i^—Mn1—N4	91.75 (8)	C31—C33—C32	118.1 (3)
N4—Mn1—N7^ii^	88.75 (8)	N8—C34—C36	107.1 (3)
N1^i^—Mn1—N5	89.60 (7)	N7—C35—N8	113.0 (2)
N5—Mn1—N7^ii^	89.44 (7)	N7—C36—C34	110.3 (2)
N1^i^—Mn1—N7^ii^	176.52 (8)	C3—C1—H1	125.00
C27—O1—C28	117.9 (2)	N1—C1—H1	125.00
C8—O2—C10	118.23 (19)	N1—C2—H2	124.00
H3A—O3—H3B	109 (2)	N2—C2—H2	124.00
Mn1—O3—H3B	118 (2)	N2—C3—H3	126.00
Mn1—O3—H3A	125.6 (18)	C1—C3—H3	126.00
H5A—O5—H5B	107 (5)	C5—C4—H4	120.00
Mn1^ii^—N1—C2	128.12 (19)	C9—C4—H4	120.00
C1—N1—C2	104.9 (2)	C5—C6—H6	120.00
Mn1^ii^—N1—C1	127.02 (16)	C7—C6—H6	120.00
C2—N2—C3	105.7 (2)	C6—C7—H7	120.00
C3—N2—C5	128.5 (2)	C8—C7—H7	120.00
C2—N2—C5	125.8 (2)	C4—C9—H9	120.00
C16—N3—C18	105.9 (2)	C8—C9—H9	121.00
C15—N3—C16	127.6 (2)	C14—C11—H11	120.00
C15—N3—C18	126.5 (2)	C10—C11—H11	120.00
Mn1—N4—C17	131.48 (16)	C10—C12—H12	120.00
Mn1—N4—C18	123.34 (17)	C13—C12—H12	120.00
C17—N4—C18	105.1 (2)	C12—C13—H13	120.00
C19—N5—C20	104.9 (2)	C15—C13—H13	120.00
Mn1—N5—C20	126.71 (16)	C11—C14—H14	120.00
Mn1—N5—C19	128.22 (16)	C15—C14—H14	120.00
C19—N6—C21	106.1 (2)	C17—C16—H16	127.00
C21—N6—C22	127.4 (2)	N3—C16—H16	127.00
C19—N6—C22	126.41 (19)	N4—C17—H17	125.00
C35—N7—C36	104.8 (2)	C16—C17—H17	125.00
Mn1^i^—N7—C36	126.59 (16)	N4—C18—H18	124.00
Mn1^i^—N7—C35	128.65 (19)	N3—C18—H18	124.00
C33—N8—C34	127.7 (2)	N5—C19—H19	124.00
C33—N8—C35	127.4 (2)	N6—C19—H19	124.00
C34—N8—C35	104.9 (2)	C21—C20—H20	125.00
C38—N9—C39	121.6 (6)	N5—C20—H20	125.00
C37—N9—C39	123.0 (6)	N6—C21—H21	127.00
C37—N9—C38	115.4 (5)	C20—C21—H21	127.00
N1—C1—C3	110.0 (2)	C22—C23—H23	120.00
N1—C2—N2	112.0 (2)	C24—C23—H23	120.00
N2—C3—C1	107.4 (2)	C23—C24—H24	120.00
C5—C4—C9	120.8 (3)	C27—C24—H24	120.00
N2—C5—C6	120.5 (2)	C26—C25—H25	120.00
N2—C5—C4	120.3 (2)	C22—C25—H25	120.00
C4—C5—C6	119.3 (2)	C25—C26—H26	120.00
C5—C6—C7	120.1 (3)	C27—C26—H26	120.00
C6—C7—C8	119.8 (3)	C32—C29—H29	120.00
O2—C8—C7	120.8 (3)	C28—C29—H29	120.00
O2—C8—C9	118.1 (3)	C31—C30—H30	120.00
C7—C8—C9	121.0 (2)	C28—C30—H30	120.00
C4—C9—C8	119.1 (3)	C33—C31—H31	120.00
O2—C10—C11	115.6 (2)	C30—C31—H31	120.00
O2—C10—C12	123.8 (2)	C29—C32—H32	119.00
C11—C10—C12	120.6 (2)	C33—C32—H32	119.00
C10—C11—C14	120.2 (2)	N8—C34—H34	127.00
C10—C12—C13	119.4 (2)	C36—C34—H34	126.00
C12—C13—C15	120.3 (2)	N8—C35—H35	123.00
C11—C14—C15	120.0 (2)	N7—C35—H35	124.00
C13—C15—C14	119.5 (2)	N7—C36—H36	125.00
N3—C15—C13	119.9 (2)	C34—C36—H36	125.00
N3—C15—C14	120.5 (2)	O4—C39—N9	123.8 (7)
N3—C16—C17	106.8 (2)	N9—C37—H37B	109.00
N4—C17—C16	109.8 (2)	N9—C37—H37C	109.00
N3—C18—N4	112.4 (2)	H37A—C37—H37B	110.00
N5—C19—N6	112.1 (2)	H37A—C37—H37C	109.00
N5—C20—C21	110.9 (2)	H37B—C37—H37C	109.00
N6—C21—C20	106.1 (2)	N9—C37—H37A	109.00
N6—C22—C23	119.4 (2)	N9—C38—H38A	109.00
N6—C22—C25	121.2 (2)	N9—C38—H38C	109.00
C23—C22—C25	119.4 (2)	H38A—C38—H38B	110.00
C22—C23—C24	120.2 (2)	H38A—C38—H38C	109.00
C23—C24—C27	120.3 (2)	H38B—C38—H38C	109.00
C22—C25—C26	119.8 (2)	N9—C38—H38B	109.00
C25—C26—C27	120.3 (2)	O4—C39—H39	118.00
C24—C27—C26	120.1 (2)	N9—C39—H39	118.00
O1—C27—C24	123.7 (2)		
			
Cl1—Mn1—N4—C17	−148.6 (3)	C19—N5—C20—C21	0.0 (3)
Cl1—Mn1—N4—C18	35.1 (2)	C21—N6—C19—N5	−0.4 (3)
O3—Mn1—N4—C17	35.4 (3)	C22—N6—C19—N5	−176.8 (2)
O3—Mn1—N4—C18	−140.9 (2)	C19—N6—C21—C20	0.4 (3)
N1^i^—Mn1—N4—C17	−53.9 (3)	C22—N6—C21—C20	176.7 (2)
N1^i^—Mn1—N4—C18	129.9 (2)	C19—N6—C22—C23	2.4 (4)
N7^ii^—Mn1—N4—C17	122.7 (3)	C19—N6—C22—C25	−179.6 (3)
N7^ii^—Mn1—N4—C18	−53.5 (2)	C21—N6—C22—C23	−173.2 (3)
Cl1—Mn1—N5—C19	135.7 (2)	C21—N6—C22—C25	4.9 (4)
Cl1—Mn1—N5—C20	−49.0 (2)	C36—N7—C35—N8	0.6 (3)
O3—Mn1—N5—C19	−48.3 (2)	Mn1^i^—N7—C35—N8	−179.58 (16)
O3—Mn1—N5—C20	127.0 (2)	C35—N7—C36—C34	0.1 (4)
N1^i^—Mn1—N5—C19	41.1 (2)	Mn1^i^—N7—C36—C34	−179.7 (2)
N1^i^—Mn1—N5—C20	−143.6 (2)	C34—N8—C33—C31	10.3 (4)
N7^ii^—Mn1—N5—C19	−135.6 (2)	C34—N8—C33—C32	−168.0 (4)
N7^ii^—Mn1—N5—C20	39.7 (2)	C35—N8—C33—C31	−173.8 (3)
Cl1—Mn1—N1^i^—C1^i^	−3.7 (2)	C35—N8—C33—C32	7.9 (4)
Cl1—Mn1—N1^i^—C2^i^	174.9 (2)	C33—N8—C34—C36	177.7 (3)
O3—Mn1—N1^i^—C1^i^	176.9 (2)	C35—N8—C34—C36	1.1 (4)
O3—Mn1—N1^i^—C2^i^	−4.6 (2)	C33—N8—C35—N7	−177.7 (2)
N4—Mn1—N1^i^—C1^i^	−97.6 (2)	C34—N8—C35—N7	−1.1 (3)
N4—Mn1—N1^i^—C2^i^	81.0 (2)	C38—N9—C39—O4	−177.6 (6)
N5—Mn1—N1^i^—C1^i^	90.3 (2)	C37—N9—C39—O4	4.0 (10)
N5—Mn1—N1^i^—C2^i^	−91.2 (2)	N1—C1—C3—N2	−0.7 (4)
Cl1—Mn1—N7^ii^—C35^ii^	2.4 (2)	C9—C4—C5—C6	0.3 (4)
Cl1—Mn1—N7^ii^—C36^ii^	−177.9 (3)	C9—C4—C5—N2	−179.8 (2)
O3—Mn1—N7^ii^—C35^ii^	−178.2 (2)	C5—C4—C9—C8	0.4 (4)
O3—Mn1—N7^ii^—C36^ii^	1.6 (3)	C4—C5—C6—C7	−0.7 (5)
N4—Mn1—N7^ii^—C35^ii^	96.2 (2)	N2—C5—C6—C7	179.4 (3)
N4—Mn1—N7^ii^—C36^ii^	−84.1 (3)	C5—C6—C7—C8	0.3 (5)
N5—Mn1—N7^ii^—C35^ii^	−91.6 (2)	C6—C7—C8—C9	0.4 (5)
N5—Mn1—N7^ii^—C36^ii^	88.2 (3)	C6—C7—C8—O2	−176.0 (3)
C28—O1—C27—C24	−11.0 (5)	O2—C8—C9—C4	175.7 (2)
C28—O1—C27—C26	170.9 (3)	C7—C8—C9—C4	−0.7 (4)
C27—O1—C28—C29	−91.0 (4)	C12—C10—C11—C14	1.4 (4)
C27—O1—C28—C30	94.8 (4)	O2—C10—C11—C14	−179.1 (2)
C10—O2—C8—C7	−80.4 (4)	C11—C10—C12—C13	−0.3 (4)
C10—O2—C8—C9	103.1 (3)	O2—C10—C12—C13	−179.7 (3)
C8—O2—C10—C11	175.7 (3)	C10—C11—C14—C15	−1.7 (4)
C8—O2—C10—C12	−4.9 (4)	C10—C12—C13—C15	−0.5 (4)
C2—N1—C1—C3	0.4 (3)	C12—C13—C15—C14	0.2 (4)
Mn1^ii^—N1—C1—C3	179.2 (2)	C12—C13—C15—N3	−178.4 (2)
C1—N1—C2—N2	0.0 (3)	C11—C14—C15—N3	179.4 (2)
Mn1^ii^—N1—C2—N2	−178.81 (16)	C11—C14—C15—C13	0.9 (4)
C3—N2—C2—N1	−0.4 (3)	N3—C16—C17—N4	0.2 (4)
C5—N2—C2—N1	179.1 (2)	N5—C20—C21—N6	−0.3 (3)
C2—N2—C3—C1	0.6 (3)	C25—C22—C23—C24	2.6 (5)
C5—N2—C3—C1	−178.8 (2)	N6—C22—C25—C26	179.7 (2)
C2—N2—C5—C4	−15.7 (4)	N6—C22—C23—C24	−179.3 (3)
C2—N2—C5—C6	164.2 (3)	C23—C22—C25—C26	−2.3 (4)
C3—N2—C5—C4	163.6 (3)	C22—C23—C24—C27	−0.9 (6)
C3—N2—C5—C6	−16.5 (4)	C23—C24—C27—O1	−179.1 (3)
C16—N3—C15—C13	−157.7 (3)	C23—C24—C27—C26	−1.2 (5)
C16—N3—C15—C14	23.7 (4)	C22—C25—C26—C27	0.2 (4)
C18—N3—C15—C13	24.1 (4)	C25—C26—C27—C24	1.5 (4)
C18—N3—C15—C14	−154.5 (3)	C25—C26—C27—O1	179.6 (3)
C15—N3—C16—C17	−179.0 (3)	O1—C28—C29—C32	−175.8 (4)
C18—N3—C16—C17	−0.5 (3)	C29—C28—C30—C31	2.2 (6)
C15—N3—C18—N4	179.2 (2)	C30—C28—C29—C32	−1.5 (6)
C16—N3—C18—N4	0.6 (3)	O1—C28—C30—C31	176.4 (3)
Mn1—N4—C17—C16	−176.5 (2)	C28—C29—C32—C33	0.3 (7)
C18—N4—C17—C16	0.2 (4)	C28—C30—C31—C33	−1.6 (6)
Mn1—N4—C18—N3	176.56 (17)	C30—C31—C33—C32	0.4 (5)
C17—N4—C18—N3	−0.5 (3)	C30—C31—C33—N8	−178.0 (3)
Mn1—N5—C19—N6	176.38 (16)	C29—C32—C33—N8	178.6 (4)
C20—N5—C19—N6	0.3 (3)	C29—C32—C33—C31	0.3 (6)
Mn1—N5—C20—C21	−176.18 (18)	N8—C34—C36—N7	−0.8 (4)

Symmetry codes: (i) *x*, *y*−1, *z*+1; (ii) *x*, *y*+1, *z*−1.

### Hydrogen-bond geometry (Å, °)

**Table:**

*D*—H···*A*	*D*—H	H···*A*	*D*···*A*	*D*—H···*A*
O3—H3A···Cl2^iii^	0.86 (2)	2.29 (2)	3.1306 (19)	166 (2)
O3—H3B···Cl2	0.85 (2)	2.27 (2)	3.093 (2)	162 (3)
O5—H5A···Cl1^iv^	0.89 (2)	2.81 (5)	3.282 (3)	114 (4)
O5—H5B···Cl1^v^	0.95 (3)	2.48 (4)	3.316 (4)	148 (5)
C17—H17···Cl2^iii^	0.93	2.67	3.553 (3)	159
C18—H18···O5^iv^	0.93	2.50	3.418 (5)	170
C23—H23···O4^vi^	0.93	2.47	3.270 (6)	145
C35—H35···Cl1^i^	0.93	2.82	3.398 (3)	121

Symmetry codes: (iii) −*x*+1, −*y*+1, −*z*+2; (iv) −*x*+1, −*y*+2, −*z*+1; (v) *x*−1, *y*, *z*; (vi) *x*, *y*, *z*+1; (i) *x*, *y*−1, *z*+1.
